# Nursing workloads in family health: implications for universal access^1^

**DOI:** 10.1590/1518-8345.0992.2682

**Published:** 2016-03-28

**Authors:** Denise Elvira Pires de Pires, Rosani Ramos Machado, Jacks Soratto, Magda dos Anjos Scherer, Ana Sofia Resque Gonçalves, Letícia Lima Trindade

**Affiliations:** 2 PhD, Full Professor, Departamento de Enfermagem, Universidade Federal de Santa Catarina, Florianópolis, SC, Brazil; 3 PhD, Adjunct Professor, Departamento de Enfermagem, Universidade Federal de Santa Catarina, Florianópolis, SC, Brazil; 4 Doctoral student, Departamento de Enfermagem, Universidade Federal de Santa Catarina, Florianópolis, SC, Brazil; 5 PhD, Adjunct Professor, Departamento de Saúde Coletiva, Universidade de Brasília, Brasília, DF, Brazil; 6 PhD, Adjunct Professor, Faculdade de Enfermagem, Universidade Federal do Pará, Belém, PA, Brazil; 7 PhD, Adjunct Professor, Centro de Educação Superior do Oeste, Universidade do Estado de Santa Catarina, Chapecó, SC, Brazil

**Keywords:** Primary Health Care, Unified Health System, Nursing, Quality Assurance, Health Care, Health Services Accessibility

## Abstract

**Objective:**

to identify the workloads of nursing professionals of the Family Health Strategy, considering its implications for the effectiveness of universal access.

**Method:**

qualitative study with nursing professionals of the Family Health Strategy of the South, Central West and North regions of Brazil, using methodological triangulation. For the analysis, resources of the Atlas.ti software and Thematic Content Analysis were associated; and the data were interpreted based on the labor process and workloads as theorical approaches.

**Results:**

the way of working in the Family Health Strategy has predominantly resulted in an increase in the workloads of the nursing professionals, with emphasis on the work overload, excess of demand, problems in the physical infrastructure of the units and failures in the care network, which hinders its effectiveness as a preferred strategy to achieve universal access to health. On the other hand, teamwork, affinity for the work performed, bond with the user, and effectiveness of the assistance contributed to reduce their workloads.

**Conclusions:**

investments on elements that reduce the nursing workloads, such as changes in working conditions and management, can contribute to the effectiveness of the Family Health Strategy and achieving the goal of universal access to health.

## Introduction

Universal access has been one of the main challenges for health systems worldwide. Currently, it has assumed so significant role that it is under discussion its inclusion as one of post-2015 millennium development goals^(^ 1 ^-^ 4 ^)^. Ensuring universal access is extremely relevant for human life and it means a great challenge for the countries and professionals of this field^(^ 2 ^-^ 3 ^,^ 5 ^)^. 

The meaning of access to the health actions and services that people need involves values such as justice and equity, which could not be incorporated into the concept of coverage. The universal coverage concept often refers to the amount of the population covered by health services, which does not mean that this number of people use the service or that the health service is able to meet the multiple and complex health needs^(^ 6 ^)^.

The work of health professionals lies in the service sector, involves a relationship between subjects of the same nature (human beings), and the work outcomes depends on collaboration between those who care and those who are cared. Furthermore, like the work of other sectors of the economy, it is heavily influenced by specific conditions under which it is performed; the manner in which it's done and under what work conditions and labor relations^(^ 7 ^)^.

The special characteristics of work in health care and the importance of workforce for its development are recognized by the Global Health Workforce Alliance and World Health Organization (WHO). In the debate on coverage and universal access, these organizations prepared a report in which they state "A universal truth: no health without a workforce"^(^ 1 ^)^. 

Therefore, to achieve the goal of universal access to health it is necessary to focus on who does the work, how it is performed and under what conditions, in different historical and social scenarios. In this sense, the analysis of the workloads present in the way of working is a promising way to guide the provision of care to users/patients, and thus improve the quality of access to health services.

Workloads are elements found in the work process that synthesize the mediation between work and wear of the worker. Workloads interact with each other and with the body of who does the work. They do not act individually, but in combination with each other and determine the condition in which the worker faces the global logic of the labor process^(^ 8 ^)^.

In Brazil, from the creation of the National Unified Health System (SUS), the principle of universality has been strengthened by defining, in the Federal Constitution, that health is a universal right and a duty of the state and one component of the quality of life. Health has been regarded as a fundamental right of the citizen and a duty of the three spheres of government. This system has the universality, equity and comprehensiveness as its doctrinal proposition and, from 1990; aims to promote changes in the hegemonic care model based on the biomedicine assumptions. The Family Health Strategy (FHS) has an important role in this scenario. 

The FHS is part of the National Primary Care Policy and adopts principles of the Primary Health Care (PHC), formulated in the World Health Organization conference, held in Alma Ata, in 1978. The PHC has been reaffirmed as strategic for universal access to health services^(^ 9 ^)^. 

The FHS has been growing significantly in Brazil and, in March 2015, 37,944 family health teams had been deployed in 5,319 municipalities, with a national population coverage of 60.56%, which represents almost 118 million people^(^ 10 ^)^.

The FHS has expanded the access to health services and strengthened the principle of universality. Nonetheless, the country is still facing serious problems to ensure universal access, equitable and comprehensive to all individuals^(^ 11 ^)^. 

The way of doing the work, as well as the complexity of the work environments and conditions available for its completion, interferes with the quality and safety of the health care results at all levels of care^(^ 12 ^-^ 14 ^)^. The nursing team represents the largest category among healthcare professionals and plays a central role in health services and in the assurance of users' safety^(^ 15 ^-^ 17 ^)^. 

In the FHS, nursing professionals are present in all teams and their work has an impact on the quality of care. Thus, to identify the workloads present in their routine helps to guide managers in the strengthening of positive factors and in the search for actions to reduce them, contributing to the viability of universal access. 

Although nursing is essential for access and operation of health services, there are still major imbalances and gaps in the availability, distribution, composition, qualification and productivity of nursing professionals, which has implications in terms of quality and safety of services provided. Excessive workloads in the daily routine of the services, worsened by poor working conditions negatively affect the results, satisfaction and health of these professionals^(^ 14 ^)^. Literature points out complex internal and external challenges to the profession as well as challenges at other levels such as historical, cultural, gender and field knowledge and fighting for appreciation of the important role that nursing plays in the health system^(^ 4 ^)^.

This study was based on the observation that health workforce is critical to achieving universal access and that the FHS is the preferred policy to achieve this goal and, in this context, nursing plays a relevant role in the healthcare sector and in the FHS. Consequently, the aim of this study was to identify the workloads of nursing professionals of the Family Health Strategy, analyzing their implications for the effectiveness of universal access **.**

For the analysis of the research results, theoretical approaches of labor process^(^ 7 ^)^ and workloads^(^ 8 ^)^ were associated.

## Method

This is a qualitative study, in which it was used methodological triangulation with data from semi-structured interviews, document analysis and observation. 

The selection of participants was based on the following inclusion criteria: nursing professionals working in the FHS teams that were distinguished by developing a good quality work, according to the information supplied by the managers; at least one municipality per region; and only FHS teams that had a core team of professionals recommended by the Ministry of Health. It was excluded those professionals who worked in the care units of mixed type. The care units of mixed type include both teams working in the Family Health Strategy teams and teams working on traditional mode. 

Data were collected from November 2010 to February 2013, in the FHS teams from three regions of Brazil: South - in three municipalities in the Southwest of Paraná; Central West - in the Federal District and North region - in the city of Belém, Pará. 

Regarding the data collection, the description of the quantity and distribution of the interviews, observation notes and documents examined are shown in Table 1. 

**Table 1:** - Distribution of data collection instruments, according to number, type and geographic region. Florianópolis, SC, Brazil, 2015

**Geographic region**	**Interviews**		**Notes**		**Documents**			
	**n**	**%**		**n**	**%**		**n**	**%**
Central West	10	43.5		17	43.6		3	17.6
North	7	30.4		10	25.6		8	47.1
South	6	26.1		12	30.8		6	35.3
Total	23	100.0		39	100.0		17	100.0

This study included 23 nursing professionals, of which nine were nurses, 13 nursing technicians and a nursing assistant, who worked in ten health teams of five municipalities in total. The number of participants was considered sufficient according to the data saturation criterion, i.e, the inclusion of new participants and teams was suspended when no new explanations, interpretations or descriptions of the phenomenon studied were found.

Among the participants, most were women (87%; n=20); aged between 30 and 49 years (61%; n=14); professional experience of at least 5 years (70%; n=16) and experience in the FHS from one to five years (61%; n=14). Regarding the employment contract, there was a strong predominance of permanently assigned employees (70%; n=16) with working hours of 40 hours per week (87%; n=20). 

Semi structured interviews followed a script that has been tested and is composed of closed and open questions. The questions aimed, primarily, to characterize the profile of the participants, and to identify the sources and implications of workloads on the professionals composing the FHS. The observations of the work context and document analysis also followed a script and the first were described in field diaries. The scripts included the information gathering on the sources of increases in workloads observed by researchers, details about the labor process and work tools, way of assisting the users/patients and documents informing about routines, physical structure and flow of care in the units.

For the analysis, it was used resources of the Atlas.ti 7.0 software and principles of the Thematic Content Analysis. In the pre-analysis stage, a storage file [Hermeneutic Unit] was created using the Atlas.ti software, in which the survey data obtained from interviews and observation [Primary Documents] was entered. A specific number was assigned to each interview [*document*]. Data exploration included the selection/trimming of the meaning units [*quotations*], creation and assignment of codes [*codes*], and these codes were grouped into categories [*families*]. Finally, during the interpretation process, it has been made associations among*quotations*, *codes* and*families*, generating display networks [*networks*], which were extracted from the software and presented in the results. It is important to emphasize that the analysis process, in all its stages, was based on a cyclical triad, namely: empirical data, theoretical foundation and perception of the researchers about the studied phenomenon. 

The ethical precepts internationally recommended for developing research involving human beings were respected. The project was approved by the Ethics Committee on Human Research of the Federal University of Santa Catarina, under protocol number 971, approved on October 25, 2010. 

The research was authorised by the institutions involved and it was asked to all participants to sign of the Informed Consent (IC). To these, it was guaranteed the right to information, to withdraw from the study at any stage and anonymity. The speeches were identified by the first letter of the professional category (N and Nt to nurse and nursing technician, respectively) followed by the first letter of the data collection region (S, C and N to designate the South, Central West and North) and by a serial number. The observation note was identified followed by the first letter of the region.

## Results

The results were sorted into two analytical categories: way of working in the FHS and increase in the workloads and way of working in the FHS and reduction of the workloads.

## Way of working in the FHS and increase in the workloads of the nursing professionals

**Figure 1:**
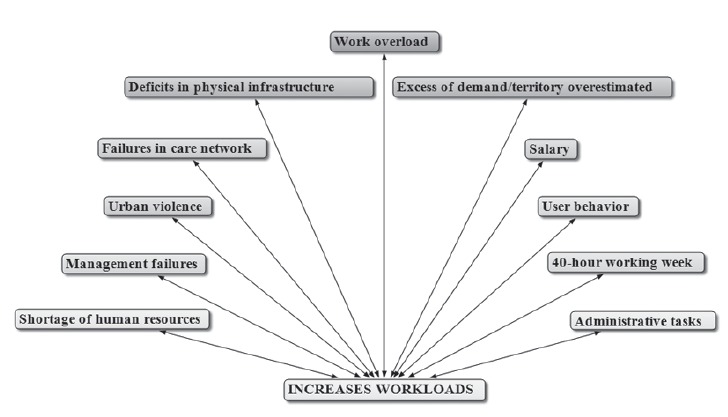
- Main elements which increase the workloads of the nursing professionals

With regard to the increase in the workloads, in all three regions of the country, the major components identified are shown in Figure 1, created by using the Atlas.ti software.

Among the main elements that increase the workloads are problems related to working conditions, which have strong association with management. Among them, it is emphasized: work overload; excess of demand; deficits in physical infrastructure; failures in the proper functioning of the care network of National Unified Health Service (SUS); dissatisfaction with the wages, considered to be insufficient, and working hours perceived as excessive; lack of qualified human resources and the overload caused by carrying out administrative tasks. 

In the three regions of the country, the workload associated with excess of demand and territory overestimated were the most significant elements for the increase in the workloads of the nursing professionals, as illustrated by the following report. 

*The workload gets intense; it´s too much service to be carried out at the same time, we often leave work so tired and still under the impression that we did not accomplish all that was under our responsibility. We have to do home care, we have to do screening, we have to carry out technical procedures within the unit, we cannot leave the doctor alone since he always needs us around and we are only two nursing technicians. If we go out to do the dressings at home, our colleague stands alone in the health unit to solve what it was already impossible to be solved by two professionals. It is inhuman to leave the colleague on his own; we go out with a guilty conscience* (NtC2).

*And we go on with the same team to assist the families of our areas and others that are discovered* (NN1).

[...] *my team is incomplete, lacking a nursing technique, I have been working only with a nurse and a doctor in my team. I am alone, so this complicates matters. The work intensifies, so we cannot do the work the way we want due to the lack of a nursing technician* (NtS2).

Failures in the care network and in the organizational and physical infrastructure have also been very significant, producing increase in the workloads, as shown in the following reports.

*Sometimes the patient's problem resolution depends on other administrative levels and then it gets jammed. Such situation frustrates us. We get to know the patient's problem and try to help, seeking ways to do it, but we know that what we could do has already been done* [...]. *If references are necessary, we know that they exist, and we seek them, but no one knows where they are.* [...] *and, besides that, these sectors are not committed to help* (NtC5).

[...] *the lack of a counter-reference* [...] *a follow up of that user/patient. I refer the patient to a specialist, to some medical specialty, but I do not get any counter-reference, so I do not know what to do* (tense)*. I know he has been to the doctor, but I do not know the further steps that should be taken* [...] *I get no feedback. It makes me very tired, very angry ... it is like an unfinished bridge*[...]*. That to me is a very bad signal* [...]. *Our infrastructure is one of the city's best, still, it is completely inadequate. In the winter, it is extremely cold and in the summer, it is too warm. This dissatisfaction has led me to use medications, I am fine now, but I used to seek answers to questions that I could not understand, I'm in therapy*(NS2).

*There is a room, without minimum requirements, where the pathology technician works. She is responsible for collecting material for the tests ordered by the medical teams. There is no sink in that room, no descartex for proper disposal of cutting and perfure materials. It was improvised a "can of milk powder" for the disposal of needles* (OBSERVATION NOTE, C).

The nursing professionals mentioned the low salaries and excessive working hours as cause of the increase in the workloads; however, salary was not mentioned in the Central West region. 

*The salary is low. Considering the stress level and the amount of work performed by the professionals* (NN1).

*The salary is not the ideal ... it is still low, but if it is compared with other colleagues from other institutions, our salary is good* (NS2).

*I am dissatisfied with the salary, there is no career path to nursing, we earn a little more because we work eight hours day, although there is no difference because it is a FHS. We work during two, four, six or more hours in the week and we get no extra pay. The working hours, eight per day, do not satisfy, it would be better to work six hours/day, this also to the user/patients,* [...] *many families are no longer followed up, which is the intention of the FHS, due to the operating schedule*(NtS1).

Problems relating to the work subject were also significant, i.e, related to the patients/users of the services. It was identified dissatisfaction of the users with the services and difficulties of access, especially in relation to the most complex services. Another triggering element of the increases in the workloads was the exposure of professionals to urban violence. Often, the care units are located in violent areas.

Way of working in the FHS and reduction of the workloads

**Figure 2:**
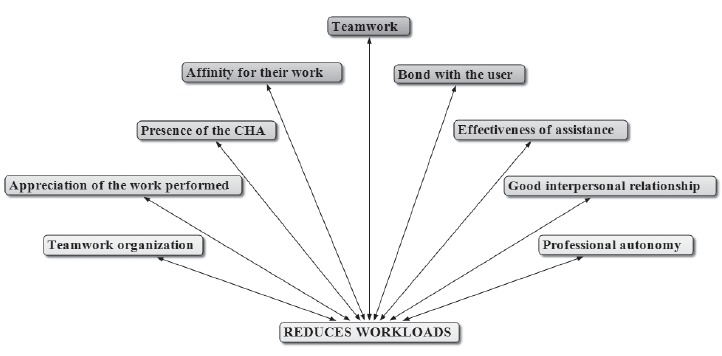
- Main elements which reduce the workload of the nursing professionals

With regard to the way of working in the FHS, considering the reduction of the workloads, teamwork has been found as the most significant, as shown in Figure 2, which was generated by the Atlas.ti software as shown below. 

Reports of nursing professionals show that teamwork, bond with the user, presence of the Community Health Agents (CHA) and affinity for their work, contribute to the reduction of the workloads. The first three components are part of what is prescribed for the FHS and are associated with the *code*"affinity for their work", suggesting that there is identity of health professionals with the care model of the FHS, which contributes to the reduction of the workloads. It also contributed to the reduction of the workloads: organization of work; respect for professional autonomy within the collective work; achieving satisfactory results; recognition of the users, colleagues and managers for the service provided, and good working relationships.

Teamwork was the main element to the reduction of the workloads. This protective aspect of teamwork is shown in the following reports. 

*Our team has a great affinity among all professionals* [...]*one supports the other; we have integration, friendship, fellowship. Working partnership. All defending the same purpose* [...]. *In addition,my coworker, the other nursing technician is very friendly at work. She does not overburden me and we do not transfer our responsability to another. We work in harmony* (NtC3).

*Teamwork is unique. Good relationship with the doctor, nursing technician and with the CHA, the power of communication, dialogue, not only with the team of which we are part, but also with the other team, so we are able do the work, because we come together, one helps the other* (NN2).

*It is positive, so we can work in partnership, all professionals*[...] *we have meetings every Friday starting at 3 p.m., we discuss a lot with the team about what has been done* [...] *from the point of view of the community, what's good and what's not, what we understand from that, what is feasible is changed. Therefore, there is an interaction, a very tight integration between the members of the team* (NS1).

In addition to teamwork, the relationship with the user and presence of the Community Health Agents are elements that contributed to the reduction of the workloads, signaling identity/affinity of the nursing professionals with the model of the FHS.

## Discussion

In this study, the results showed a predominance of situations that increase the workloads of the nursing professionals. Among the elements that contributed to the increase of the workloads, are several elements related to the working conditions and management of the health system, of which the FHS is part. 

The problem of working conditions has been repeatedly recognized as a serious problem in the health field in Brazil and, particularly in nursing, including deficits in the quantity and quality of the workforce, excessive working hours, deficits in the salary and problems in the work environment^(^ 14 ^-^ 15 ^)^. Deficits in working conditions negatively influence the health of the nursing professionals and results of the assistance provided by these professionals^(^ 13 ^-^ 18 ^)^.

With regard to the work overload, even considering the involvement of subjective aspects, it was identified that there is a discrepancy between demand and team's ability. Other aspects related to working conditions, identified in this research, indicate that there are unsatisfactory objective conditions, which interact with each other resulting in the increase in the workloads.

Overloaded nursing professionals that, in addition, perform their work in adverse conditions tend to suffer damage to their health, which increases the work absenteeism, resulting in greater overload on those who remain. This situation negatively influence the effectiveness and quality of results in health care. The service might be available and even represent that formally there is coverage, however, the population that uses the service faces barriers and limited or inefficient care. 

Another factor that contributed to the increase in the workloads were the problems in the relationship of nursing professionals with users, in particular due to their unmet needs and insecurity of these professionals to act in areas of violence.

Increased workloads cause dissatisfaction, wear and even illness on who does the work, and difficulty to perform a creative and effective work, which interferes in the possibilities to ensure access, from the perspective of a comprehensive care. 

Universal access is related to providing assistance to the entire population at all levels of the system^(^ 6 ^-^ 19 ^)^, considering the complexity of the needs in health. It includes the environment where the work is carried out, method of organizing work and how care is provided, as well as the relationship established between professionals and users. It is worth mentioning that health work is highly dependent on labor force^(^ 1 ^)^, therefore any initiative in this field requires focusing on who performs the work. 

The absence of geographic, economic, socio-cultural, organizational or gender barriers has been recognized as required for universal access^(^ 4 ^)^. In this study, it was identified economic, socio-cultural and organizational barriers in the PHC scenario in the country, which has generated increased workloads of the nursing professionals and hampered the effectiveness of the FHS as a potentiating policy of universal access. 

On the other hand, the elements that contribute to the reduction of the workloads, found in this study, show that there is an affinity of the nursing professionals with this kind of care model. Among these aspects are the teamwork, establishment of bond between professionals and users and the presence of the CHA, which are the main elements of the FHS. 

Factors that contribute to the reduction of the workloads can contribute to the permanence of professionals in this care model, since they are also positive for a closer approximation with the way of working required in the FHS. FHS is inspired by the PHC, internationally recognized as a strategy for achieving the universal access to health^(^ 20 ^)^. 

The identity with the work and search for recognition and appreciation of the work, reinforce the capacity to act of the collective, offering prospects of coping of the adverse side of the work contexts. To believe that work has been carried out in the "right way" promotes satisfaction and, even in inappropriate situations, helps in the reduction of the workloads. 

In Brazil, the definition of universal right to health and access to health services has ocurred in a scenario of valuation of the citizens' rights, which occurred during the implementation of the Federal Constitution in 1988. The current challenge relates to dealing with complex problems arising from the demographic and epidemiological transition and insufficient public investment in health^(^ 3 ^)^. Greater public investment in health can contribute to improving working conditions, the operation of the network and hence universal access. FHS integrates the SUS as a privileged strategy for its accomplishment; however, the everyday of services has increased the workloads of the teams, which complicates their work, as well as the resolution of the problems of the users.

## Conclusion

FHS adopts the principles of the PHC internationally recognized as central in the search for equity and universal access. In this research, the nursing professionals recognized these precepts as positive. However, deficits in the working conditions and in the health system management have adversely affected the workloads, which hinders the effectiveness of the FHS. Increased workloads, particularly the overload on the nursing professionals, affect the efficiency and quality of care, the way the user is assisted and cared in the health services and consequently, the quality of access. 

In the contexts where the research was conducted, aspects that increase the workloads contribute to hinder the access to the health services from the perspective of universality, equity and resolution. The role prescribed for the family health teams is extremely ample, requiring longitudinality in care, polyvalent knowledge and professional training for an intersectoral intervention. 

The research results show that it is necessary to invest in strategies that strengthen the teamwork, professional autonomy of nursing professionals and incorporation of technologies that contribute to the efficacy of care and reduction of the work overload. These strategies may help to empower these professionals for their daily routine, as well as to participate politically in the management of the healthcare services and social control, aiming at the implementation of measures to strengthen the profession and improve universal access.

These results also contribute to the development of the nursing as a healthcare profession and as a discipline of scientific knowledge by providing knowledge about the work of these professionals within the scope of the most significant public policies prevailing in Brazil, which corresponds to the priority strategy of the WHO to universal access.

It is worth mentioning that this study was carried out with an intentional sample, which limits its generalization. However, it is important to note that even intentionally including teams considered as of good quality, deficits in the working conditions and in aspects related to the system management were significant. These findings suggest that more studies in this same perspective might be promising for further investments in health and advancement of knowledge in nursing and health.
